# Occurrence and fate of pharmaceuticals, personal care products (PPCPs) and pesticides in African water systems: A need for timely intervention

**DOI:** 10.1016/j.heliyon.2022.e09143

**Published:** 2022-03-18

**Authors:** Charles Obinwanne Okoye, Emmanuel Sunday Okeke, Kingsley Chukwuebuka Okoye, Daniel Echude, Felix Attawal Andong, Kingsley Ikechukwu Chukwudozie, Henrietta Ukamaka Okoye, Chigozie Damian Ezeonyejiaku

**Affiliations:** a Ecology and Environmental Biology Unit, Department of Zoology and Environmental Biology, University of Nigeria, Nsukka, 410001, Nigeria; b Biofuels Institute, Jiangsu University, Zhenjiang, 212013, China; c School of Environment and Safety Engineering, Jiangsu University, Zhenjiang, 212013, China; d Natural Science Unit, School of General Studies, University of Nigeria, Nsukka, 410001, Nigeria; e Department of Biochemistry, University of Nigeria, Nsukka, 410001, Nigeria; f Entomology Unit, Department of Zoology and Environmental Biology, University of Nigeria, Nsukka, 410001, Nigeria; g Department of Microbiology, University of Nigeria, Nsukka, 410001, Nigeria; h Social Policy Unit, Department of Public Administration and Local Government, University of Nigeria, Nsukka, 410001, Nigeria; i Bioconservation Unit, Department of Zoology, Nnamdi Azikiwe University, Awka, 5025, Nigeria; j Organization of African Academic Doctors (OAAD), Off Kamiti Road, 25305000100, Nairobi, Kenya

**Keywords:** Emerging contaminants, Remediation, African water systems

## Abstract

The occurrence of emerging contaminants (ECs) such as pharmaceuticals, personal care products (PPCPs) and pesticides in the aquatic environment has raised serious concerns about their adverse effects on aquatic species and humans. Because of their toxicity and bioactive nature, PPCPs and pesticides have more potential to impair water systems than any other contaminants, causing several adverse effects, including antibiotic resistance, reproductive impairment, biomagnification, bioaccumulation, etc. Over 35 publications from Africa have reported on the occurrence and fate of PPCPs and pesticides in African water systems with little or no data on remediation and control. As a result, adequate intervention strategies are needed for regulating the persistence of PPCPs and pesticides in African water systems.

## Graphical abstract

**Figure:**
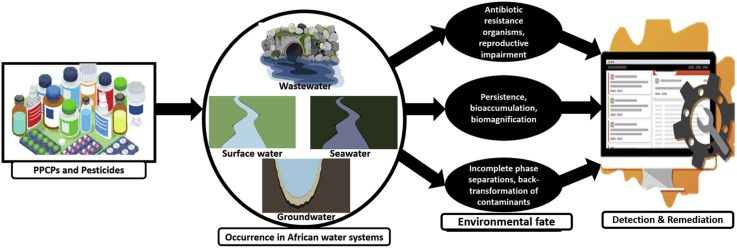

## 1 Introduction

The recurring exculpation of emerging contaminants (ECs) in the environment has raised serious concerns about their adverse effects on humans and aquatic species [1, 2, 3] and the development of antibiotic resistance due to pharmaceuticals, personal care products (PPCPs), and pesticides being released into the environment [4]. PPCPs and pesticides refer to any product used for either personal health or cosmetic purposes and any product used in the agricultural industry to maintain health or promote plant and animal development. Particularly, PPCPs and pesticides comprise a diverse collection of thousands of chemical substances, including prescription and over-the-counter therapeutic drugs for humans and animals, vitamins, and other nutritional supplements, herbicides, biopharmaceuticals, diagnostic agents, cosmetics, and fragrances, and growth-enhancing chemicals used in livestock operations [3]. PPCPs and pesticides are used in various human activities, obliging their discharge into the environment distinctively. They have transformed the present-day living standard, and their usage is fundamental to the environment [5, 6]. In most of these regularly used items, medications, and pesticides, chemical components may persist in sewage systems and eventually enter the aquatic environment as metabolites or modified substances. Because of their bioactive nature and harmful toxic metabolites, PPCPs and pesticides are thought to have more potential to impair water systems than any other contaminants [7]. Over 35 publications from Africa (Table 1) have reported on the occurrence and fate of various types of PPCPs and pesticides, compared to about 730 and 143 from Europe and the United States, respectively [8]. However, most studies on the prevalence of PPCPs are biased towards developed countries, with the majority of them focusing on their concentrations, fate, and behavior. Although these compounds have been found in various environments, recent reviews have focused on the presence of PPCPs and pesticides in Africa, with a particular focus on the aquatic system [5, 9, 10].

**Table 1:** Studies on the occurrence and fate of PPCPs and pesticides in various water systems in Africa.

Region	Occurrence	PPCPs and pesticides type	Concentration (*μ*g L^−1^)	Detection	Fate	References
Southern Africa	Surface water	Ibuprofen	19.2	HPLC-DAD	Reduces sperm motility and fertilization, influences the hatch rate, motion, locomotion, and gene expression in aquatic organisms	[52]
Southern Africa	Wastewater	Ibuprofen	1.38	HPLC-MS/MS	Incomplete phase separations, resistant to biodegradation	[26]
West Africa	Surface water/groundwater	Dieldrin	1.51	GC-ECD	Bioconcentration and biomagnification via terrestrial and aquatic food chains; increase the water solubility of nonpolar compounds	[13]
West Africa	Surface water	Dieldrin, endrin, dichlorodiphenyltrichloroethane (DDT), endosulfanaldehyde, and phosphomethylglycine	0.02–0.15	GC-ECD	Biomagnification via terrestrial and aquatic food chains	[37]
North Africa	Ground water, surface water, wastewater	Ibuprofen, naproxen, ketoprofen, diclofenac	0.1109–6.554	GC-MS	Higher toxicity of direct discharge of untreated wastewaters, Implicates removal efficiencies of wastewater treatment plants, pseudo-persistence	[19]
West Africa	Surface water	Trimethoprim, Tetracycline, Acetylsalicylic acid, Betasitosterol, Bezafibrate, Chlortetracycline, Clarithromycin, Clofibric acid, Doxycycline, Estradiol, Estriol, Estrone, Etofibrate, Fenofibrate, Fenoprofen, Ibuprofen, Indometacin, Ketoprofen, Mestranol, Pentoxifylline, Phenacetin, Phenazone, Sulfadiazine, Sulfadimidine	0.01–8.84	GC-MS	Biomagnification via terrestrial and aquatic food chains	[54]
West Africa	Surface water, groundwater	Hydrocortisone, Acetaminophen, Metformin, Gabapentin, Nicotine, Codeine, Sulfamethoxazole, Caffeine, Trimethoprim, Amoxicillin, Tramadol, Metoprolol, Propranolol, Carbamazepine, Erythromycin, DEET, Oxazepam, Mefloquine, Naproxen, Valsartan, Diazepam, Glyburide, Diclofenac, Ibuprofen, Clotrimazole, Meclofenamic acid, Gemfibrozil	0.003–1.614	LC-MS/MS	Development of antibiotic-resistant genes in bacterial populations	[14]
West Africa	Surface water, wastewater	Equilin, Caffeine, Chloramphenicol, Erythromycin, Ciprofloxacin, Roxithromycin, Sulfamethoxazole, Lincomycin, Acetamidophenol/paracetamol/acetaminophen, Carbamazepine, Diclofenac sodium, Oxybenzone, N, N-dimethyl3-methylbenzamide (DEET), Triclosan, Triclocarban, Sulfathiazole, Methylparaben	0.001–0.47	LC-MS/MS	Biomagnification via terrestrial and aquatic food chains	[55]
West Africa	Surface water, seawater	Acetaminophen, Amoxicillin	0.0058–1.23	HPLC	Biomagnification via terrestrial and aquatic food chains	[42]
West Africa	Groundwater	Sulphadoxine, Amodiaquine, Chloroquine	58–451.6	HPLC	Persistence and bioaccumulation in the food web	[56]
West Africa	Surface water	Oxytetracycline	0.003–0.0048	HPLC	Creation of antibiotic-resistant strains in natural bacterial populations	[57]
Southern Africa	Surface water, wastewater	Ciprofloxacin, Aspirin, Ampicillin, Nalidixic acid, Ketoprofen, Bezafibrate, Diclofenac, Ibuprofen, Acetaminophen, Sulfamethoxazole, Atenolol, Caffeine, Streptomycin, Tetracycline, Erythromycin, Chloramphenicol, Tylosin	0.14–0.97	HPLC-DAD	Development of antibiotic resistance and feminization or masculinization of aquatic organisms, pseudo-persistence	[58, 59]
East Africa	Surface water, wastewater	Sulfadoxin, sulfamethoxazole, paracetamol, ibuprofen, sulfamethoxazole, methylparaben, carbamazepine, diclofenac, indomethacin	100–1000	UPLC-MS/MS	Persistent, and tend to accumulate, limited biodegradability, easily attached to a particle for transport in water, bioaccumulate in aquatic organisms	[36]
West Africa	Seawater	Metronidazole, Trimethoprim, Sulphamethoxazole, Ampicillin, Cloxacillin	0.07–1.599	HPLC	Development of antibiotic-resistant genes in bacterial populations	[60]
East Africa	Wastewater	Amoxicillin, ampicillin, and ciprofloxacin	37–367	HPLC-UV	Incomplete removal during soil passage due to incomplete sorption	[61]
North Africa	Surface water	Oxycladine citrate, nepagine, and salbutamol	0–4.7.0		Persistent, and tend to accumulate, limited biodegradability, easily attached to a particle for transport in water, bioaccumulate in aquatic organisms	[62]
North Africa	Surface water	Amoxicillin, erythromycin, sulfamethoxazole, tetracycline, ciprofloxacin, oxolinic acid, trimethoprim	0.0019–4.107	LC–MS/MS	Development of antibiotic-resistant strains in natural bacterial populations	[18]
Northeastern Africa	Surface water, groundwater	Bisphenol A (BPA), methylparaben, ethylparaben, propylparaben, butylparaben, o-phenylphenol	0.0064–0.071	UPLC–MS/MS	Elicits adverse effects in reproductive organs of aquatic organisms, bioaccumulation, and biomagnification in the food web	[63]
North Africa	Surface water	Acetaminophen, ibuprofen	0.22–0.9	HPLC-UV	Development of antibiotic-resistant strains in natural bacterial populations	[64]
North Africa	Seawater, groundwater	Chloramphenicol, thiamphenicol, florfenicol, paromomycin, dihydrostreptomycin, kanamycin, apramycin, streptomycin, amikacin, sisomicin, neomycin, gentamycin	3.4–18.4	UPLC-MS/MS	Persistence of antibiotic-resistant microorganisms	[65]
North Africa	Groundwater, wastewater	Carbamazepine, carbamazepine epoxide, dihydroxycarbamazepine	0.0102–0.1145	HPLC	Highly recalcitrant to standard bioremediation; wastewater leaches into groundwater depending on its sorption potential and on its transformation into the soil	[46]
Southern Africa	Seawater	Diclofenac, sulfamethoxazole, phenytoin, carbamazepine, lamivudine, caffeine, acetaminophen	0.010–0.034	UPLC TQ-MS	Poses an adverse environmental risk to non-targeted organisms via ​biomagnification ​in the food chain	[41]
North Africa	Wastewater	Carbamazepine, naproxen, ibuprofen	8.02–132	HPLC-UV	Incomplete phase separations, resistant to biodegradation	[66]
Southern Africa	Surface water, wastewater	Ibuprofen, ketoprofen, diclofenac, naproxen, triclocarban, triclosan, codeine, tramadol, atenolol, chloramphenicol, ciprofloxacin, clarithromycin, tetracycline, acetaminophen, norfloxacin, ofloxacin, sulfamethoxazole, sulfasalazine, azithromycin, trimethoprim, caffeine, dextromethorphan, mephedrone, methamphetamine, cocaine, carbamazepin, cotinine, nicotine, alkylphenol, ethoxylates, fluoxetine, fexofenadine	0.0276–0.4502	UPLC/TQD-MS	Back-transformation of contaminants, recurring negative mass balances, decreases efficiency, capacity, and selectivity of contaminant sorption	[53]
Southern Africa	Wastewater	Naproxen and ibuprofen, triclosan	10.7–127.7	HPLC-UV	Renders traditional sampling approaches insufficient	[67]
Southern Africa	Surface water, wastewater	Ciprofoxacin, ofoxacin, norfoxacin, tetracycline, atenolol, triclosan, triclocarban, diclofenac, acetaminophen, ibuprofen, ketoprofen	0.3–119	LC- TQ-MS	Low removal rate impedes the growth and survival of aquatic organisms in receiving water bodies	[68]
North Africa	Groundwater, wastewater, surface water,	Atenolol, benzafibrate, 1-H-benzotriazole, bisphenol A, caffeine, carbamazepine, diclophenac, ethylparabene, fenofibric acid, furosemide, gemfibrozil, ibuprofene, ibuprofene, ibuprofene, ketoprofene, methlyparabene, metoprolol, naproxen, nonylphenol, o-desmethyl-naproxene, oxazepam, paracetamol, propylparabene, sulfamethoxazole, tolyltriazoles, triclocarban, triclosan, trimethoprim, antibiotic	<0.289	HPLC, UPLC/MS-MS	Incomplete removal during soil passage due to incomplete sorption and/or biodegradation, high oxygen demand, photodegradation of leads to harmful disposal in surface waters	[69]
Southern Africa	Surface water, wastewater	Efavirenz, emtricitabine, lamivudine, nevirapine, ritonavir, zidovudine, 8,14-dihydroxyEfavirenz, 12-hydroxy-Nevirapine, desthiazolylmethyloxycarbonyl ritonavir, Nevirapine-D	<0.172	LC-MS/MS	Significant losses of polar targets in WWTPs due to low solubility and partial removal, ​eventual seepage ​to ​surface ​water and ​groundwater	[70]
East Africa	Surface water	Sulfamethoxazole, trimethoprim, ciprofloxacin, lamivudine, nevirapine, zidovudine	0.509–13.8	LC-ESI-MS/MS	Development of antimicrobial resistance and possible toxicity to sensitive organisms	[71]
Southern Africa	Groundwater	Atrazine, carbamazepine, cinchonidine, cinchonine, diphenylamine, enilconazole, ephedrin, flecainide, fluconazole, hexazinone, imidacloprid, metazachlor, metolachlor, minoxidil, nalidixicacid, paracetamol, phenytoin, sebuthylazine-desethyl, simazine, sulphisomidine, tebuthiuron, telmisartan, temazepam, terbumeton, terbuthylazine, thiabendazole	<0.35	LC-MS/MS	Low sperm volume and motility, fetal growth defects, increase in DNA damage, congenital anomalies, and cardiovascular	[20]
Southern Africa	Wastewater	Efavirenz, nevirapine	5.5–14.0	GC-MS	De-conjugation of metabolites in the WWTP, resistance to degradation, lack of binding of the metabolites to the sludge	[72]
Southern Africa	Surface water, wastewater	Zalcitabine, tenofovir, abacavir, efavirenz, lamivudine, didanosine, stavudine, zidovudine, nevirapine, indinavir, ritonavir, lopinavir, caffeine	0.0265–0.43	LC-MS/MS, UHPLC-MS/MS	Resistant to degradation, ubiquitous occurrence to surface water, promotes the development of drug resistance in other pathogens	[73]
Southern Africa	Surface water	Efavirenz, nevirapine, carbamazepine	0.164–0.593	LC-MS/MS	Induces antibacterial resistance, neurobehavioral disorders in aquatic animals, diminished predator evasive behavior, less aggressive nest defense	[74]
Southern Africa	Wastewater, groundwater	Penciclovir, famciclovir, ribavirin, paracetamol, ketoprofen, diclofenac, fenoprofen, ibuprofen, carbamazepine, primidone, sulfamethoxazole, pindolol	<0.0196	HPLC- CAD	Inefficient removal in WWTPs, distribution by aqueous transport, food-chain dispersal, mineralization to carbon dioxide and water, adsorption on suspended solids	[75]

CAD-charged aerosol detector; TQ-MS- triple quadrupole mass spectrometer; GC-ECD-gas chromatography – electron capture detector; LC/MS/MS- liquid chromatography-tandem mass spectrometry; HPLC- high-performance liquid chromatography; UHPLC/MS/MS- ultra high-performance liquid chromatography-tandem mass spectrometry; UPLC- ultra performance liquid chromatography; ESI- electrospray ionization.

Recently, PPCPs and pesticides have been discovered in African waters and are mainly deposited in sediments and microplastics [2, 11]. Despite momentous improvements in science and technology over the past decades, the fate of these contaminants, as they enter into the aquatic environment, remains somewhat unsettled, and awareness of the surging detection and associated ecotoxicological impacts of these emerging contaminants in the aquatic environment is increasing [5, 6]. Modern analytical techniques uncover more rapid, sensitive, and simplified analyses of emerging contaminants in water systems [12]. For example, Okoya et al. [13]and Ebele et al. [14] described a Gas Chromatography – Electron Capture Detector (GC-ECD) and Liquid Chromatography with tandem mass spectrometry (LC/MS/MS) method to analyze several classes of PPCPs and pesticides in surface water groundwater and wastewaters in the West African region. The detection of PPCPs and pesticides have been reported at concentrations ranging from parts-per-trillion (ng L^−1^) to parts-per-billion (μg L^−1^) in various water systems [5, 8, 15, 16, 17]. However, most of these studies vary as some focus on specific compounds and concentrations, while some studies do not focus on the particular kind of these ECs. Besides, the compounds' nature varies between them [18, 19, 20, 21]. The occurrence and fate of PPCPs and pesticides in water bodies such as surface waters, seawaters, wastewaters, and groundwater have been the subject of few studies in Africa (Table 1), although they are the largest category of ECs [21, 22, 23]. Because some of these contaminants are not easily degradable, it is critical to monitor the behavior of PPCPs and pesticides in most developing nations, such as Africa, where waste disposal is primarily through landfills.

Moreover, they can contaminate surface water, the ocean, and, most importantly, groundwater, which is a crucial water source for a vast section of the population [4, 14]. PPCPs and pesticides enter groundwater directly in the form of treated and untreated wastewater and through landfill leachate, animal wastes, domestic sewage, and contaminated surface water [21, 24]. Most PPCPs and pesticides alter physiology and biochemical processes in humans, plants, and animals. Their occurrence in the groundwaters indicates contamination through direct disposal of residual medicines in the landfills, farmlands, and household waste or body excretions [25]. Therefore, it is essential to study the occurrence and fate of these contaminants in various water systems because contamination patterns may differ by habitat or region [26]. Hence, this review presents the available information on the occurrence and fate of PPCPs and pesticides in African water systems, emphasizing the need for eliminating these contaminants from water using various remediation technologies.

## 2 Sources, transport, and routes of exposure of PPCPs and pesticides

**Figure 1:**
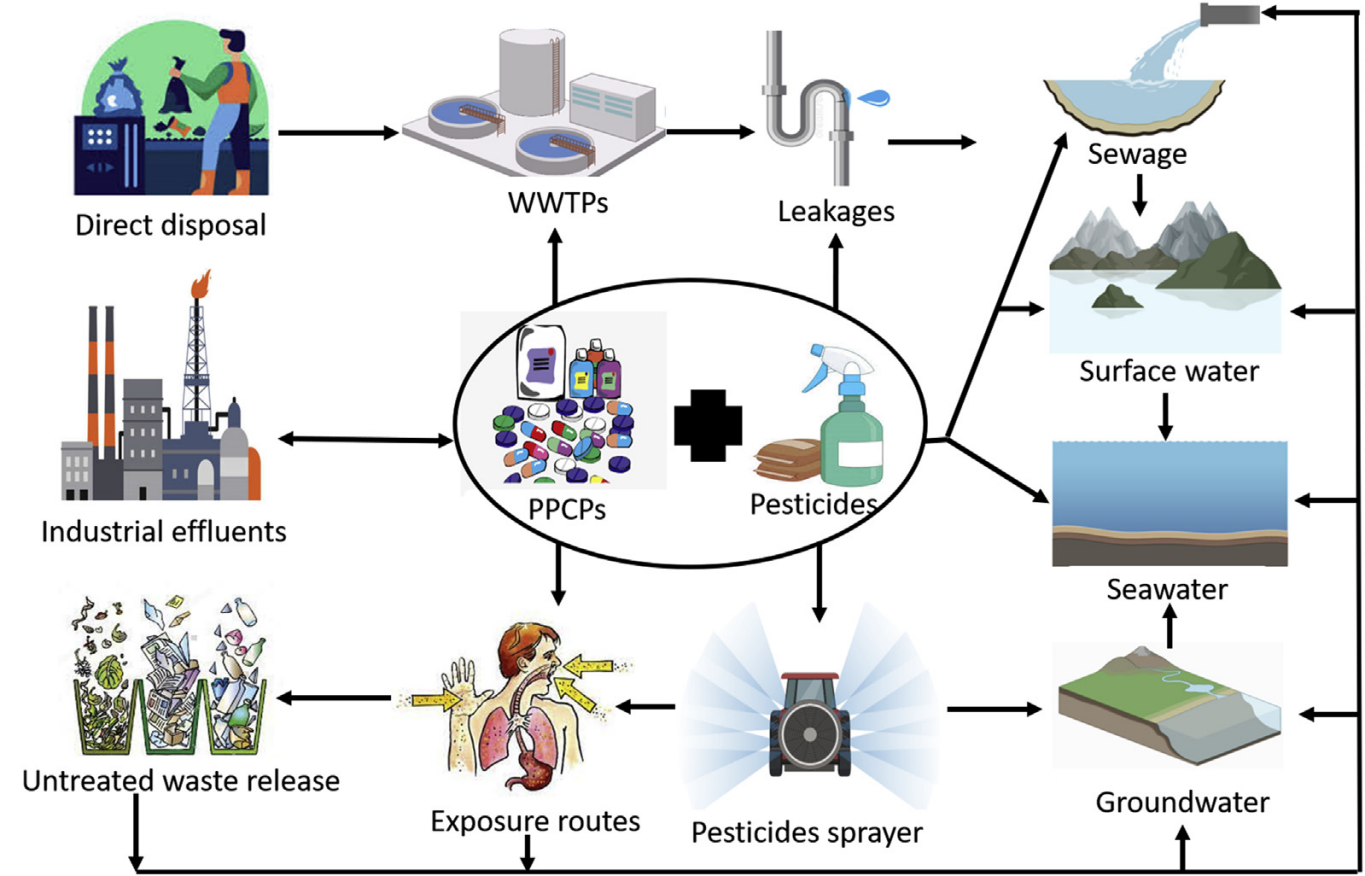
Sources, transport, and exposure routes of PPCPs and pesticides in various water systems. Contamination from specific point sources is due to direct disposal and untreated releases from domestic, commercial, industrial leakages, spills on farmyards, and wastewater treatment plant effluents. Source: authors.

PPCPs and pesticides are released globally from either a point or diffused sources, including landfill leachate, effluents, combined sewer overflows, treated sewage sludge, animal feedlots, aquaculture, and agricultural lands [27], and they enter the environment through a number of pathways from several activities and actions (Figure 1). The main routes of PPCPs and pesticides to the environment are primarily WWTPs effluent (to water systems) and, secondarily, terrestrial run-off (to soil). Moreover, the pace of the movement and severity of these contaminants depends on the quality of water treatment, compounds physicochemical properties, compound concentration, species affected, and their fate in the ecosystem, concomitantly, depends on the characteristics of the receiving environment/species [3]. The main route of exposure to PPCPs is through the excretion to the sewage system following use and manufacturing activities [4]. Besides, exposure to pesticides is mainly from oral contact.

In contrast, human contact occurs through a variety of routes, including (i) inhalation by breathing mobile pesticides, such as during on-farm pesticide spraying, (ii) dermal, and (iii) ocular routes, such as during accidental splashing or spraying pesticides on unprotected skin/eyes of agricultural workers and pesticide industry workers [28, 29]. PPCPs, such as drugs, soaps, detergents, toothpaste, pharmaceuticals, medical equipment, plastics, and textiles, contain various chemicals. Humans are exposed to these chemicals through a variety of routes, including absorption (e.g., soaps, toothpaste), ingestion (e.g., drinking water, medications, food), inhalation (e.g., aerosols, dust), and injection/implantation, as a result of their common and extensive use (e.g., medical sutures and devices) [30]. Moreover, releases to surface waters from wastewater treatment systems, aquaculture facilities, and field run-off, as well as releases from manure application, are all considered in regulatory environmental risk assessment techniques for PPCPs and pesticides [31].

Considering the variation in the management and usage practices of PPCPs and pesticides in Africa and other parts of the world, a major exposure pathway in one climatic region may be less relevant in another. Also, because population connectivity to wastewater treatment technology is limited in various parts of the world, regulatory exposure modeling will not always be appropriate. However, a better understanding of PPCPs and pesticides release mechanisms and prevailing exposure pathways in various African countries is required.

## 3 Occurrence and fate of PPCPs and pesticides in various water systems in Africa

### 3.1 Surface waters

PPCPs and several pesticides have been identified as potential environmental hazards in a variety of freshwater environments. Little is known in the African freshwater environment regarding the occurrence, fate, and behavior of PPCPs and pesticides [23, 32, 33, 34, 35]. Pesticides and PPCPs contamination of the freshwater systems can occur in various ways. For example, a critical pathway is applying pesticides on agricultural land and absorbing the PPCPs by the body following usage of drugs and other products and excretion and release into the sewage system. High concentrations of PPCPs ranging from 10 to 84.60 μg L−1 have been reported in the surface waters of South Africa and Kenya [26, 36]. Many of these compounds exceed their acceptable concentration limits, highlighting the importance of understanding the fate and transport of these compounds in freshwater environments. Several different PPCPs, including ibuprofen, naproxen, caffeine, ketoprofen, and diclofenac, have been reported to be dominant in Algerian rivers [21]. These PPCPs showed high-risk concentrations, with a severe ecological impact on daphnia and algae [23]. The high percentage of organic pesticides such as dieldrin endrin, dichlorodiphenyltrichloroethane (DDT), endosulfanaldehyde, and phosphomethylglycine present in the Oluwa, and Owan rivers in Nigeria, has been described as contaminants of environmental concern, considering the rising accumulation potential of these compounds in the food chain [13, 37]. The high polarity of dieldrin causes increased affinity for organic matter resulting in bioaccumulation in the food chain [38]. Organochlorine pesticides accumulate in fish samples in the Niger River, indicating their extreme toxicity and persistence. These compounds bioaccumulate and have the potential for long-term transport, resulting in approximately 200,000 deaths globally from lifetime consumption of pesticide-contaminated fish, with a higher number from developing countries [17, 37, 38]. Accumulation of PPCPs and pesticides in surface waters leads to lifelong adverse impacts on aquatic life, such as changes in growth rate, behavior, reproduction, and modifications at the biochemical level in primary producers up to secondary consumers [7, 39].

### 3.2 Seawaters

The degree of salinity differs significantly between freshwater and seawater, with seawater being naturally saltier and heavier than freshwater. The density of seawater increases with depth, which helps keep ECs suspended in the water column [40]. Seawater is in a constant state of motion. It must be checked regularly for pollutants in marine species, which can only arise from long-term contact with sewage-contaminated water [41]. Unfortunately, no study has reported the presence of pesticides, and very few studies have been done on the occurrence of PPCPs in African seawater. A recent study reported the presence of different PPCPs, including amoxicillin and methylparaben, higher than their environmental concentrations in Lagos Lagoon, Nigeria [42]. Because PPCPs have low volatility and are highly polar and hydrophilic, they will predominantly spread across the environment via water transport and the marine food chain. The examination of numerous PPCPs in various portions of edible fish species from Kalk Bay harbor, South Africa, demonstrated that these chemicals pose a significant danger to pelagic fish, aquatic organisms, including humans who consume them [43]. The presence of PPCPs in seawater, such as caffeine and antibiotics, is a good indication of fecal contamination. Caffeine is released into the environment in its natural state from the human digestive system via feces. Furthermore, disinfectants and antibiotics are known to generate selection for resistance in the gene pool of microbes, eventually rendering them immune to antibiotics or antimicrobial agents [41].

### 3.3 Groundwaters

In Africa, groundwater is a vital water source with crucial environmental concerns since it provides water for human consumption, irrigation, and ecosystem demands. Anthropogenic groundwater pollution is hazardous to human and environmental health and well-being [44]. Various organic ECs, which occur in considerable quantities owing to recent and previous human activities, are primarily contaminating groundwater around the planet [45]. The fate of these contaminants depends largely on their capacity to sorb onto soil and aquifer materials during infiltration [46]. A very high concentration (0.3–15.6 μg L^−1^) of diclofenac has been reported in South African surface water above the acceptable limit (0.1 μg L^−1^) proposed by the European Union (EU) watch list. Because groundwater is replenished from surface water and used for direct drinking purposes, the possibility of ECs in surface water could be a threat [47].

### 3.4 Wastewaters

Studies have established a priority list for the regulatory framework for future treatment and monitoring programs in Africa based on the incidence and concentration levels of different contaminants in wastewaters [47]. Pesticides and PPCPs have been found in wastewater released into surface water worldwide at quantities exceeding 100 μg L^−1^ [48, 49, 50, 51]. Matongo et al. [26] reported the presence of a high concentration of antipyretic ibuprofen (117 μg L^−1^) in wastewater samples from South Africa. PPCPs, including ibuprofen, diclofenac, and caffeine, are used in treating common symptoms like fever, pain, and inflammation in humans. Approximately 10 percent of their consumption is excreted in an unmetabolized form, which may be their possible pathway to wastewaters [19, 47, 52]. The resulting back-transformation of PPCPs and pesticides have been reported during wastewater treatment with recurring negative mass balances in wastewater treatment plants (WWTPs) [53].

## 4 Analytical methods for the detection of PPCPs and pesticides in water systems

PPCPs and pesticides have been found in the aquatic environment worldwide due to their widespread consumption and improper disposal. Conventional water/wastewater treatment techniques are insufficient for removing them, resulting in their accumulation in the receiving aquatic environment and the potential for harm to the ecosystem and human health [76]. The African populace indiscriminately uses PPCPs and pesticides, and some of them are excreted as metabolites, with sewage being the most common cause of their release into the environment. These compounds are in almost all water systems (wastewater, surface water, groundwater, etc.) at concentrations ranging from ng/L to μg/L [77]. The isolation and extraction of ECs from water are based on solid-phase extraction (SPE) and other methods, including dispersive microextraction by sorbent, ultrasound-assisted extraction, solid-phase extraction, pressurized hot water extraction, SPE using multicartridges, etc. [78, 79, 80]. Recently several methods have been developed for the detection of PPCPs and pesticides at low concentrations including, high-performance liquid chromatography (HPLC), triple quadrupole mass spectrometer (TQ-MS), ultra-high-performance liquid chromatography-tandem mass spectrometry (UHPLC/MS/MS), gas chromatography/mass spectrometry (GC/MS) and GC/MS/MS, LC–electrospray tandem MS (LC–ES/MS/MS) [79]. These instruments allow a highly efficient separation to be achieved with highly sensitive and selective detection.

Moreover, the use of complex methods is desirable because it allows a reduction of cost and time and offers global patterns of determination with a single analysis. These methods easily facilitate an eco-friendly analysis. Sample preparation becomes the central part of the analysis in these multi-residue methods, affecting all from sample collection and storage to the specific instruments used for final quantification. However, the analytes' polarity influences the choice of chromatographic method for analyzing the final extract [79].

a. Liquid chromatography-electrospray tandem MS (LC–ES/MS/MS)

Liquid chromatography-mass spectrometry (LC-MS) is the prevailing technique for detecting PPCPs and pesticides because LC offers a versatile and universal separation mechanism suitable for non-gas chromatography (GC) amenable and the majority of GC-amenable compounds [81]. Generally, compounds with polar characteristics are more suitable for LC, and those with nonpolar properties are more amenable to GC. Besides, most PPCPs are polar or moderately polar. Moreover, the need to deal with more polar pesticides is one of the main reasons for choosing LC-MS/MS over GC-MS [82]. Because of apparent advantages like less sample pretreatment and the ability to detect polar or thermally stable chemicals, LC techniques have largely supplanted GC [83].

b. Gas chromatography/mass spectrometry (GC/MS)

Gas chromatography is used more often than liquid chromatography due to the polarity of PPCPs and pesticides and, in many cases, the necessity to perform a chemical conversion of analytes into volatile derivatives before GC analysis. However, the method quantification limits are lower in the case of GC use. For example, in the detection of diclofenac and carbamazepine. Besides, there is no correlation between sample volume and method detection limit values [84]. GC-MS remains a popular methodology since it is still considered a highly efficient separation technique, but lengthy sample derivatization processes are often required to ensure analyte volatility [51].

c. High-performance liquid chromatography (HPLC)

High-performance liquid chromatography has enabled the detection of several environmental contaminants that are highly polar or nonvolatile with high molecular weight. Based on the lowest and highest calibration standards usually utilized, instrumental analysis using the HPLC technique detects PPCPs and pesticides concentrations ranging from 0.005 to 1.0 μg per liter. The reporting levels for this method are compound dependent and have been experimentally determined based on the precision of quantitation of compounds from water samples in single-operator experiments [85]. In addition, HPLC coupled with quadrupole time-of-flight–tandem mass spectrometry (Q-TOF–MS) has been used to profile wastewater composition and evaluate the water pollution markers belonging to emerging contaminants [86]. The quadrupole–time-of-flight (Q-TOF) tandem mass spectrometer is currently one of the most selective devices coupled with liquid chromatography. Moreover, it is characterized by a very high separation efficiency [87].

d. Ultra-performance liquid chromatography (UPLC) and UHPLC- ultra-high-performance liquid chromatography

UPLC and UHPLC have gained importance in analyzing PPCPs and pesticides, and many studies have employed this technique. It saves time and solvent consumption without altering or improving sensitivity and peak resolution [88]. Pesticides including cyanazine, simazine, atrazine, and promethazine have been detected using ultra-high-performance liquid chromatography coupled with quadrupole time-of-flight–tandem mass spectrometry (UPLC-QTOF-TMS). The results revealed a linear range of 6–600 ng/ml at 0.01–0.04 and 0.04–0.15 ng/ml detection and quantification limits [87]. UHPLC has emerged as a powerful approach, mainly due to its ability to directly transfer existing high-performance liquid chromatography (LC) conditions. Presently, PPCPs and pesticides from Kenyan rivers were analyzed using UHPLC coupled to mass spectrometry, and the results revealed the concentrations of antiretrovirals, antibiotics, and pesticides prevalent in effluent water at 1 μg L−1 [36].

e. Triple quadrupole mass spectrometer (TQ-MS)

A triple-quadrupole mass spectrometer is a tandem MS method in which the first and third quadrupoles operate as mass filters, while the second induces fragmentation of the analyte through interaction with a collision gas. It is a radiofrequency-only quadrupole that may be employed in scan mode. The approach can be used to quantify or collect structural information. Product ion scan, precursor ion scan, and neutral loss scan are standard sequences for structural mass spec, followed by selective reaction monitoring or multiple reaction monitoring. Increased selectivity, lower quantitation limits, a larger linear range, and improved accuracy are some of the benefits [51, 89].

f. Electrospray ionization (ESI)

Electrospray ionization is an essential method of molecule ionization used to analyze multi-component mixtures in an LC-MS system. Electrospray ionization (ESI) and electron impact are two types of ion sources routinely employed (EI). For GC-MS instruments, EI is by far the most popular ionization method. This ionization source was used in almost all of the GC-MS techniques. Nonetheless, because it ionizes molecules straight from the liquid phase, ESI is now the most extensively utilized ionization technique in chemical and biochemical analysis for liquid form samples [51]. This soft ionization source employs electrical energy to aid the transport of ions from solution to gaseous phase without fragmentation [90]. Moreover, the pervasive body of knowledge about the occurrence and fate of PPCPs and pesticides in African water systems establishes that ESI is the most widely used coupled to LC devices [51].

## 5 Remediation technologies for PPCPs and pesticides in water systems

PPCPs and pesticides have been discovered in various water matrices, including surface water, groundwater, sewage, and treated effluents. Some of these contaminants are related to substantial ecological consequences even at trace amounts [91, 92]. Although there is increased global environmental concern about emerging organic pollutants such as PPCPs and pesticides, limited information is available on their remediation in Africa [5, 36]. Several factors, including physicochemical properties of targeted compounds and operating conditions of the processes in the WWTPs, influence the removal efficiency of PPCPs and pesticides in wastewater. These contaminants are removed or retained in WWTPs after entering the wastewater. Recently, wastewater stabilization ponds (WSPs) have been adopted to swiftly remove PPCPs and pesticides in Kenya. The WSPs reported a high potential with removals up to 99% efficiency. Various water remediation technologies have been explored for various PPCPs and pesticides, including physicochemical process, biological process, and advanced oxidation processes (AOPs) [93, 94]. However, there are significant differences among different removal methods and classes of contaminants in water systems [95]. These technologies display a wide range of variability of removal efficiency for different ECs across various water systems.

## 6 Physicochemical process

This process includes several treatment techniques, including filtration and ultrafiltration treatment techniques, activated carbon treatment, photolysis, coupled treatment, and ultrasonication.

### 6.1 Filtration and ultrafiltration

Ceramic fine ultrafiltration membrane is efficient for treating and removing anti-inflammatory, calming, antibiotic, antibacterial, and antifungal substances from water matrices. Filtration techniques facilitate the treatment of water samples by removing suspended substances, such as suspended particles, colloids, and microorganisms, from samples to prevent obstruction of the cartridges or significant interferences in subsequent treatment processes [5, 96].

### 6.2 Activated carbon treatment

This involves the use of phase-changing technologies utilizing activated carbon (AC) such as adsorption by entrapped activated carbon in alginate, biosorption in a baffled duckweed pond system, adsorption through porous sugarcane bagasse activated carbon (SCB-AC), and the removal of analgesic, antibiotics, and anti-inflammatory substances from water [97, 98]. AC is commonly utilized in this process because of its high specific surface area and porosity. For instance, AC derived from wood eliminates over 90% of acetaminophen, but AC derived from herbaceous plants removes 60–87% of the same ECs [99].

### 6.3 Photolysis

This includes direct photolysis and indirect photolysis and has been reported for successfully removing anti-infectives such as sulfamethoxazole and antibiotic such as trimethoprim in effluents [5].

### 6.4 Coupled treatment

This process includes applying the Fenton-biological process to remove antibiotics and anti-inflammatory substances like paracetamol, diclofenac sodium, and associated by-products [100]. This process is effective for degrading and mineralizing various ECs.

### 6.5 Ultrasonication

This process is a sophisticated treatment technology recently discovered for eliminating complex inorganic and organic pollutants from water and wastewater. It is characterized by low secondary emissions, safety, and energy savings. Besides, multiple reaction zones with variable amounts of reactive oxygen species and temperatures are formed during ultrasonication, including a gaseous zone, a gas-liquid interphase zone, and a bulk liquid zone [101]. However, the creation and collapse of bubbles occur in the bulk liquid zone due to the cavitation process. As a result, ultrasonication operations result in the oxidation and annihilation of various contaminants with diverse physicochemical properties [101, 102].

## 7 Biological process

This process involves the application of microbial communities in the WSPs and biofilms, biodegradation under aerobic and anaerobic conditions, and an activated sludge process. The effective removal of female sex hormones, analgesic/anti-inflammatory drugs, and steroids from water samples has been reported using these techniques [103]. However, various biological processes are available, with activated sludge systems being the most popular for treating ECs due to their performance. Moreover, aerobic or anaerobic methods can be used in conjunction with other secondary treatment processes depending on the type of contaminant. Other biological processes such as soil filtration and biological filtration have been investigated to remove ECs with interesting results, despite activated sludge being the most prevalent. The key challenge in applying biological processes for the removal of ECs is the inadequacy of precise analytical techniques that can identify and quantify these compounds in complex matrices. Further research into the development of extraction techniques for the isolation and quantification of ECs in activated sludge and/or other biological process by-products is possible because of this knowledge gap [104].

## 8 Advanced oxidation processes (AOPs)

In recent years, attention to AOPs has led to the rapid development of their improved ability to remove contaminants compared to traditional water treatment techniques. AOPs are regarded the promising technologies for removing pollutants from water, with broad applicability, little competition for pollutant degradation, and high mineralization efficiency [101]. The generation of hydroxyl radicals, the primary property of AOPs, has been linked to the high removal rates. The procedures in the AOPs group have distinct routes for producing free radicals and different work conditions and materials [104]. This process involves photo-electrocatalytic oxidation at photoanode (FTO (∗)/BiVO4/BiOI) under visible light for the removal of analgesic and antibiotic [105].

Therefore, applying these remediation technologies for different types of PPCPs and pesticides could purposively address significant factors, including the concentration of contaminants, dose, density, temperature, pH, time, and effect of catalysts ultrasonication power and degradation efficiency of the treatment processes. Nevertheless, serious needs to remediate the water systems from several contaminants have prompted researchers from different parts of the world to exploit polymer-based adsorbents with the ability to adsorb multiple contaminants simultaneously in the shortest possible time [106].

## 9 Conclusion and prospects

Emerging contaminants like PPCPs and pesticides have been reported in African water systems and have persisted due to incomplete removal and resistance to breakdown. PPCPs and pesticides have resulted in several adverse effects, including antibiotic resistance, reproductive impairment, biomagnification, bioaccumulation, etc. As a result, more investigation on the occurrence, fate, transport, and behavior of PPCPs and pesticides in African water systems is urgently needed to understand contamination thoroughly and prevent its harmful effects. Moreover, adopting the use of remediation technologies is required to reduce and possibly mitigate the persistence of these emerging contaminants. Besides, risk assessment of PPCPs and pesticides is critical for minimizing aquatic contamination and, ultimately, human health. Therefore, the substantial assortment of PPCPs and pesticides data in African water systems is crucial for understanding the need for effective monitoring and control of these contaminants.

## Declarations

### Author contribution statement

All authors listed have significantly contributed to the development and the writing of this article.

### Funding statement

This research did not receive any specific grant from funding agencies in the public, commercial, or not-for-profit sectors.

### Data availability statement

Data included in article/supplementary material/referenced in article.

### Declaration of interests statement

The authors declare no conflict of interest.

### Additional information

No additional information is available for this paper.
